# WRKY Transcription Factors Involved in Activation of SA Biosynthesis Genes

**DOI:** 10.1186/1471-2229-11-89

**Published:** 2011-05-19

**Authors:** Marcel C van Verk, John F Bol, Huub JM Linthorst

**Affiliations:** 1 Institute of Biology, Leiden University, Sylvius Laboratory, Sylviusweg 72, 2333 BE Leiden, The Netherlands

**Keywords:** WRKY28, WRKY46, ICS1, PBS3, salicylic acid, plant defense, signal transduction, transcription factors

## Abstract

**Background:**

Increased defense against a variety of pathogens in plants is achieved through activation of a mechanism known as systemic acquired resistance (SAR). The broad-spectrum resistance brought about by SAR is mediated through salicylic acid (SA). An important step in SA biosynthesis in Arabidopsis is the conversion of chorismate to isochorismate through the action of isochorismate synthase, encoded by the *ICS1 *gene. Also *AVR*_*PPHB *_*SUSCEPTIBLE 3 *(*PBS3*) plays an important role in SA metabolism, as *pbs3 *mutants accumulate drastically reduced levels of SA-glucoside, a putative storage form of SA. Bioinformatics analysis previously performed by us identified WRKY28 and WRKY46 as possible regulators of *ICS1 *and *PBS3*.

**Results:**

Expression studies with *ICS1 promoter::β-glucuronidase *(*GUS*) genes in *Arabidopsis thaliana *protoplasts cotransfected with *35S::WRKY28 *showed that over expression of WRKY28 resulted in a strong increase in GUS expression. Moreover, qRT-PCR analyses indicated that the endogenous *ICS1 *and *PBS3 *genes were highly expressed in protoplasts overexpressing WRKY28 or WRKY46, respectively. Electrophoretic mobility shift assays indentified potential WRKY28 binding sites in the *ICS1 *promoter, positioned -445 and -460 base pairs upstream of the transcription start site. Mutation of these sites in protoplast transactivation assays showed that these binding sites are functionally important for activation of the *ICS1 *promoter. Chromatin immunoprecipitation assays with haemagglutinin-epitope-tagged WRKY28 showed that the region of the *ICS1 *promoter containing the binding sites at -445 and -460 was highly enriched in the immunoprecipitated DNA.

**Conclusions:**

The results obtained here confirm results from our multiple microarray co-expression analyses indicating that WRKY28 and WRKY46 are transcriptional activators of *ICS1 *and *PBS3*, respectively, and support this *in silico *screening as a powerful tool for identifying new components of stress signaling pathways.

## Background

Because of their sessile nature, plants have evolved very sophisticated mechanisms to actively cope with different sorts of stresses. The various defense mechanisms are controlled by signaling molecules like salicylic acid (SA), jasmonic acid (JA), and ethylene, or by combinations of these signal compounds. SA accumulates locally in infected leaves, as well as in non-infected systemic leaves after infection with biotrophic pathogens and mediates the induced expression of defense genes, resulting in an enhanced state of defense known as systemic acquired resistance (SAR) [1-5]. SAR is a long-lasting broad-spectrum resistance against a variety of pathogenic fungi, bacteria and viruses [6,7]. Also exogenous application of SA results in induced expression of defense related genes [8,9]. Among the genes that are induced during SAR is a set of genes collectively known as PR (pathogenesis-related) genes, with members encoding anti-fungal β-1,3-glucanases (PR-2), chitinases (PR-3, PR-4) and PR-1, which are often used as molecular markers for SAR [7,9-11].

Genetic studies have revealed important components of the SA signal transduction pathway, briefly outlined as follows: After perception of pathogen attack by cytoplasmic TIR-NB-LRR receptors, several genes are involved in initiation of the defense response. One of these genes is *ENHANCED DISEASE SUSCEPTIBILITY 1 *(*EDS1*), which is probably activated after elicitor perception [12]. EDS1 heterodimerizes with *PHYTOALEXIN DEFICIENT 4 *(*PAD4*) and their nuclear localization is important for subsequent steps in the signaling pathway [13,14]. Both *EDS1 *and *PAD4 *are induced by pathogen infection and SA application. Another enhanced disease susceptibility gene (*EDS5*) that is also situated upstream of SA biosynthesis is expressed at high levels upon pathogen infection in an *EDS1- *and *PAD4*-dependent manner [15]. The *eds5 *mutant plants are no longer able to accumulate high levels of SA upon pathogen infection and are unable to initiate the SAR response [16].

Biosynthesis of SA can occur via two different pathways, the pathway that synthesizes SA from phenylalanine [17], and the isochorismate pathway. Inhibition of the phenylalanine pathway still allows accumulation of SA [18,19]. An important step in the isochorismate pathway is the conversion of chorismate to isochorismate (ICS). Expression of a bacterial *ICS *gene in plants causes accumulation of SA, constitutive expression of *PR *genes and constitutive SAR [20], whereas the *sid2 *mutant corresponding with a defective *ICS1* gene, is compromised in accumulation of SA and unable to mount SAR [16,21]. Expression of the *ICS1 *gene is rapidly induced after infection [21]. AVR_PPHB _*SUSCEPTIBLE 3 *(*PBS3*), of which the pathogen-induced expression is highly correlated with expression of *ICS1*, is acting downstream of SA. In the *pbs3 *mutant, accumulation of SA-glucoside and expression of *PR-1 *are drastically reduced. The *PBS3 *gene product is a member of the auxin-responsive GH3 family of acyl-adenylate/thioester forming enzymes of which some have been shown to catalyze hormone-amino acid conjugation, like the protein encoded by the *JAR1 *gene that catalyzes the formation of JA-isoleucine. However, the observation that PBS3 is not active on SA, INA and chorismate leads to the hypothesis that PBS3 must be placed upstream of SA [22-24].

Although many mutants have been reported to affect SA accumulation, no direct transcriptional regulators of genes like *ICS1 *or *PBS3 *have been identified. For *ICS1 *the presence of many TGAC core sequences, as present in the binding sites for WRKY transcription factors, has been hypothesized to be important for transcriptional regulation of *ICS1 *gene expression [25]. Here we describe two WRKY transcription factors that were previously identified in our group via a bioinformatics analysis to be closely co-expressed with *ICS1 *and *PBS3*. Co-expression analyses in protoplasts showed that WRKY28 and WRKY46 positively regulated the expression of *ICS1 *and *PBS3*, respectively. In addition, the binding sites for WRKY28 in the *ICS1 *promoter were identified.

Our results indicate that WRKY28 and WRKY46, which themselves are both rapidly induced by pathogen elicitors [26,26], link pathogen-triggered defense gene expression to the accumulation of SA via induction of *ICS1 *and *PBS3 *gene expression.

## Results

### WRKY28 Activates *ICS1::GUS *Gene Expression in Arabidopsis Protoplasts

**Figure 1:**
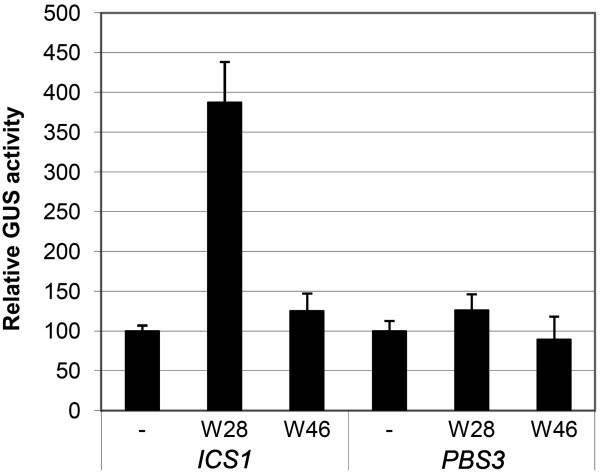
**Transactivation of *ICS1 *and *PBS3 promoter::GUS *reporter genes by WRKY28 and WRKY46 in Arabidopsis protoplasts**. The fusions contained promoter sequences of 960 bp and 1000 bp upstream of the transcription start sites of the *ICS1 *or *PBS3 *genes, respectively. Protoplasts were transfected with 6 μg of vector pRT101 containing *35S::WRKY28 *(W28) or *35S::WRKY46 *(W46) inserts, or with the empty vector (minus sign). The left three bars, correspond to the protoplasts co-transfected with 2 μg of the *ICS1::GUS *construct, the right three bars, to protoplasts co-transfected with 2 μg of the *PBS3::GUS *gene. The bars represent the average relative GUS expression observed in four experiments. GUS expression induced in the presence of the empty pRT101 vector was taken as 100%. Error bars represent the SEM.

The co-expression analysis from van Verk *et al. *[28] indicated that WRKY28 and WRKY46 could play a role in regulation of *ICS1 *and *PBS3*. To verify that WRKY28 and WRKY46 can act as positive transcriptional regulators of *ICS1 *and/or *PBS3 *gene expression we performed transactivation assays in Arabidopsis protoplasts. Protoplasts were cotransfected with plasmids containing either the *WRKY28 *or *WRKY46 *coding region behind the *35S *promoter, together with a plasmid containing the GUS reporter gene cloned behind the 1 kb promoter region of *ICS1 *or of *PBS3*. As controls, the *promoter::GUS *fusions were cotransfected with an "empty" plasmid lacking the *WRKY28 *or *WRKY46 *coding region. The results of these transactivation assays are shown in Figure 1. *ICS1 *promoter-directed GUS expression is increased approximately 4-fold by WRKY28 in comparison to the empty vector control. No increase is observed after cotransfection with the WRKY46 plasmid. In the case of *PBS3 *promoter-directed GUS expression, neither WRKY28 nor WRKY46 positively stimulated gene expression.

**Figure 2:**
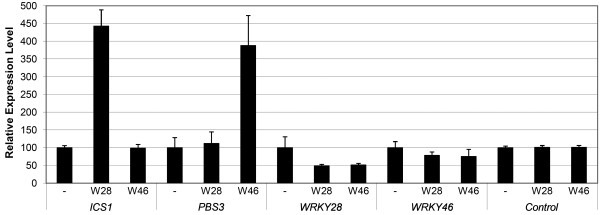
**Effect of WRKY28 and WRKY46 on the expression of endogenous Arabidopsis genes**. Expression of *ICS1*, *PBS3*, *WRKY28*, *WRKY46 *and four household genes in Arabidopsis protoplasts was measured by qRT-PCR. Expression of each gene was measured in protoplasts transfected with the empty pRT101 vector (minus sign) or with the pRT101 vector containing *35S::WRKY28 *(W28) or *35S::WRKY46 *(W46) expression constructs. Bars represent the average level of mRNA accumulation observed in three experiments. mRNA levels in protoplasts transfected with the empty pRT101 vector were taken as 100%. The control represents the average of the data obtained with the four household genes. Error bars represent the SEM.

To analyze the effect of WRKY28 and WRKY46 on expression of endogenous *ICS1 *and *PBS3 *genes, Arabidopsis protoplasts were transfected with *35S::WRKY28 *or *35S::WRKY46 *plasmids and incubated overnight, after which total RNA was isolated for qRT-PCR analysis of the expression of the endogenous *ICS1 *and *PBS3 *genes. Often, WRKYs positively regulate their own expression [29] and therefore expression of the endogenous *WRKY28 *and *WRKY46 *genes was also investigated. The constitutive housekeeping genes *Actin3*, *Actin7*, *Actin8 *and *β-Tubelin *were used as controls. The results of the qRT-PCR analyses are shown in Figure 2. WRKY28 overexpression resulted in a 4.5 fold increase of *ICS1 *mRNA. This suggests the presence of WRKY28 responsive elements in the *ICS1 *promoter, at least part of which are present in the 1 kb fragment analyzed in Figure 1. WRKY28 did not increase expression of the *PBS3 *gene. Apparently, neither the 1 kb fragment of the *PBS3 *promoter (Figure 1) nor the full-length promoter contains WRKY28 responsive elements. Overexpression of WRKY46 had no effect on expression of the *ICS1 *gene, indicating that the full-length promoter of this gene does not contain WRKY46 responsive elements. However, WRKY46 overexpression resulted in a 4-fold increase of *PBS3 *mRNA accumulation. This suggests that the *PBS3 *promoter contains WRKY46 responsive elements, located more than 1 kb upstream of the transcription start site. Obviously, there is no positive effect of WRKY28 or WRKY46 on the expression of the corresponding endogenous WRKY genes, but both WRKYs did have a slightly negative effect on the expression of the endogenous *WRKY28 *gene.

### Characterization of the WRKY28 Binding Sites in the *ICS1 *Promoter

**Figure 3:**
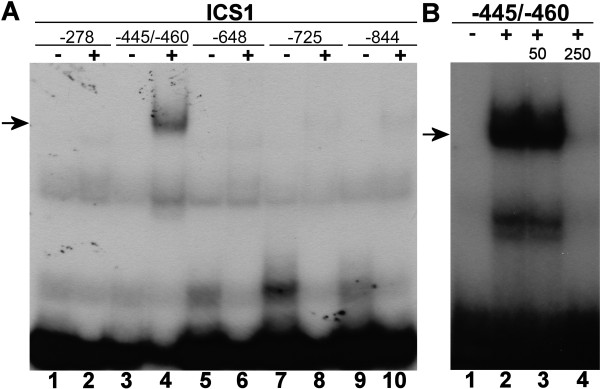
**Binding of WRKY28 to *ICS1 *promoter fragments**. (A) EMSAs were performed with promoter fragments of 30 bp, each containing a TGAC core sequence (positions -278, -445/-460, -648, -725) or a WK-like box (-844) in the center. The location of these sequences in the *ICS1 *promoter relative to the transcription start site is given above the lanes. (B) EMSAs were performed with a 30-bp fragment of the *ICS1 *promoter containing TGAC core sequences at position -445 and -460. The EMSAs in panel B were done without addition of unlabeled competitor DNA, or in the presence of a 50-fold or 250-fold excess of unlabeled competitor DNA as indicated above the lanes. The promoter fragments were incubated with recombinant GST/WRKY28 fusion protein (plus-signs) or without this protein (minus-signs). The position of protein-DNA complexes is indicated by an arrow.

WRKY proteins are generally considered to bind to the consensus W-box sequence TTGAC(C/T) [30]. The 1 kb *ICS1 *promoter does not contain a true W-box, although a number of TGAC core sequences is present (positions -725, -648, -460, -445 and -278). Furthermore, a WK-like box (TTTTCCA) that resembles the WK-box TTTTCCAC identified by van Verk *et al. *[31] is present at position -844. As a first step towards the characterization of WRKY28 binding sites in the *ICS1 *promoter, we prepared 30-bp promoter fragments that contained a TGAC core sequence or the WK-like box in the center. (The two inverted TGAC sequences at positions -445 and -460 were present in one 30-bp fragment.) After labeling, the fragments were assayed for their ability to bind to a purified glutathione S-transferase (GST)/WRKY28 fusion protein expressed in *E. coli*, using electrophoretic mobility shift assays (EMSAs). The results of EMSAs with these fragments as probes are shown in Figure 3A. The shifted band in Lane 4 indicates that the 30-bp fragment containing the two cores at -445 and -460 was bound to the GST/WRKY28 fusion protein. With none of the other WK-like or W-box core sequences a shift was observed (Figure 3A, Lanes 2, 6, 8, 10). To verify the binding specificity of the 30-bp fragment containing the TGAC cores at positions -445 and -460, competition experiments were done with 50- and 250-fold excess unlabelled fragments (Figure 3B). Evidently, addition of a 250-fold excess unlabelled fragment completely outcompeted the binding to the probe (Figure 3B, Lane 4), indicating that this *ICS1 *promoter fragment specifically interacted with WRKY28.

**Figure 4:**
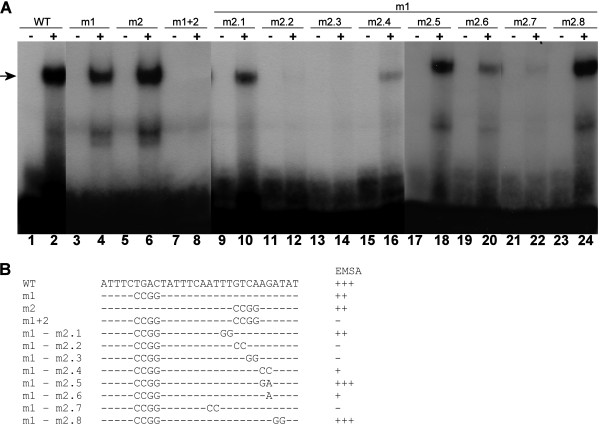
**Binding of WRKY28 to mutated *ICS1 *promoter fragments**. (A) EMSAs were performed with annealed 30-bp oligonucleotides containing the *ICS1 *promoter region indicated as -445/-460 in the legend of Figure 3 with mutations as indicated in panel B. Plus signs above the lanes indicate binding mixtures containing 0.5 μg recombinant GST/WRKY28. Minus signs above the lanes indicate binding mixtures without recombinant protein. The position of the protein-DNA complexes is indicated by an arrow. Plus and minus signs in panel B indicate the relative abundance of the shifted probe.

We speculated that the two TGAC core sequences at -445 and -460 could be binding sites for WRKY28 and set out to further investigate which site is responsible for the observed shift. Therefore, a scanning analysis was performed with a series of annealed complementary oligonucleotide probes in which the coresequences were changed to CCGG (Figure 4B, m1, m2 and m1+2). The results of EMSAs with these fragments are shown in Figure 4A, Lanes 1 to 8. Mutation of either the core at -460 (m1) or at -445 (m2) does not abolish binding of WRKY28 to the fragment (Figure 4A, compare Lanes 2, 4 and 6). However, mutation of both cores in mutant m1+2 disrupts binding (Figure 4A, Lane 8). This suggests that both binding sites are equally important.

To further analyze the requirements for binding of WRKY28, pairwise mutations of the sequence around the core at -445 were scanned in an m1 background (Figure 4B). The results are shown in Figure 4A, Lanes 9 to 24. Mutations m2.1 and m2.4 show binding to WRKY28 (Figure 4A, Lanes 10 and 16). As expected, mutations within the core sequence completely abolished binding of WRKY28 (m2.2 and m2.3, Figure 4A, Lanes 12 and 14). Since the TGAC core at -460 has TC upstream of the core and the inverted core at -445 has a CT in this position, we checked to which extend the T or C nucleotides are important for binding. Changing CT to TC resulted in a binding of WRKY28 that was as strong as to the wild type sequence (m2.5, Figure 4A, Lane 18). Changing CT to TT significantly lowered binding (m2.6, Figure 4A, lane 20), suggesting that the presence of a C at either position -1 or -2 from the core is important for binding WRKY28. We further analyzed the effect of mutations at positions -3/-4 and +3/+4 from the core. Pairwise mutation of nucleotides at -3/-4 did not alter the binding of WRKY28 (m2.8, Figure 4A, Lane 24), however no shift was observed when the nucleotides at +3/+4 were mutated, indicating that this flanking sequence is important for binding of WRKY28 (m2.7, Figure 4A, Lane 22).

**Figure 5:**
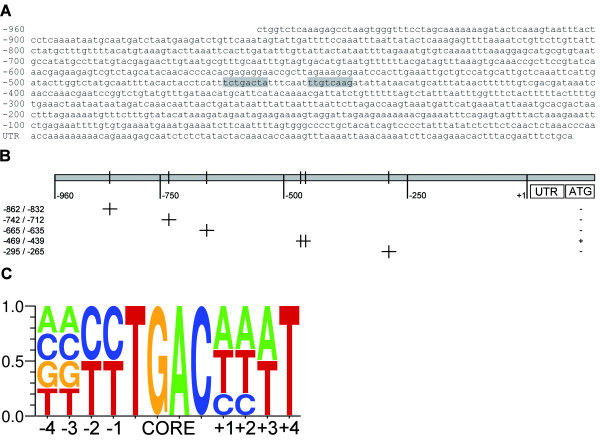
**Summary of Electrophoretic Mobility Shift Assays with WRKY28**. The indentified WRKY28 binding sites are indicated against a grey background in the sequence of the 960 bp *ICS1 *promoter (A). Schematic representation of the *ICS1 *promoter fragments analyzed by EMSA (B). Plus-signs in the right column indicate fragments that produced band shifts; minus-signs, fragments that did not produce a band shift. The position of the WK-like sequence or TGAC core sequences is indicated by vertical lines. Consensus WRKY28 binding sequence deduced from the EMSAs (C).

To summarize the results of the EMSAs, Figure 5A shows the 960 bp *ICS1 *promoter with the characterized WRKY28 binding sites indicated against a grey background. A schematic representation of the fragments tested in EMSAs for binding WRKY28 is given in Figure 5B. Figure 5C shows the consensus binding sequence with an essential C at either the -1 or -2 position, which was generated using the program WebLogo [32] by combination of the characterized binding sites and the results of the mutational analysis of the binding site at -445.

### Mutational Analysis of WRKY28-Mediated Activationof *ICS1::GUS *Gene Expression in Arabidopsis Protoplasts

**Figure 6:**
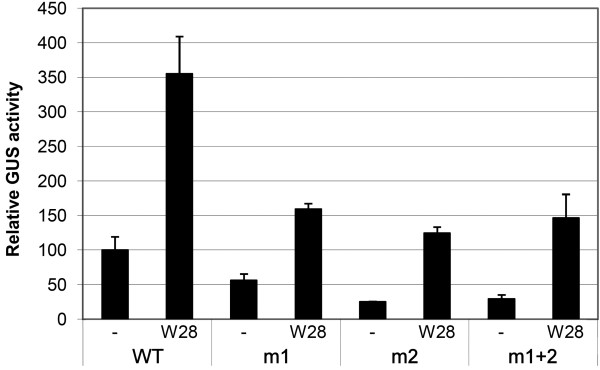
**Transactivation of *ICS1::GUS *genes with mutations in WRKY28 binding sites**. Protoplasts were transfected with 2 μg of wild-type *promoter::GUS *constructs or *promoter::GUS *constructs containing the mutations m1, m2 or m1+2 as indicated in Figure 4B. W28, cotransfection with 6 μg of expression vector pRT101 containing *35S::WRKY28*. Minus signs, cotransfection with 6 μg of empty expression vector. The bars represent the percentage of GUS activity from triple experiments relative to that of the protoplasts cotransfected with the *promoter::GUS *construct and an empty expression vector, which was set to 100%. Error bars represent the SEM.

The results from the transactivation assays, qRT-PCR and EMSA experiments indicate that WRKY28 plays a role in inducible *ICS1 *gene expression. To more directly demonstrate that the binding sites at positions -460 and -445 are involved in WRKY28 activation of *ICS1 *gene expression, Arabidopsis protoplasts were cotransfected with a WRKY28 expression plasmid together with a plasmid containing the GUS reporter gene cloned either behind the 960 bp wild-type *ICS1 *promoter or behind *ICS1 *promoters with the m1, m2 and m1+2 mutations as indicated in Figure 4B were introduced in the 1 kb *ICS1 *promoter and their effects studied in cotransfection experiments in Arabidopsis protoplasts. The results of these transactivation assays are shown in Figure 6. While cotransfection of *35S::WRKY28 *with the wild-type *ICS1 promoter::GUS *increased GUS expression approximately 3.5-fold in comparison to the basal level obtained in protoplasts cotransfected with the empty vector, expression dropped significantly with promoter constructs containing the m1 or m2 mutation (Figure 6). Combination of m1 and m2 (m1+2) did not lower GUS expression more than the single mutations (Figure 6). This result supports the notion that WRKY28 activates *ICS1 *expression through specific binding sites in the promoter at -445 and -460 bp upstream of the transcription start site.

### Chromatin Immunoprecipitation Analysis

The transactivation experiments in protoplasts and the *in vitro *binding studies described above support a role for WRKY28 as a transcriptional activator of *ICS1*. To check if WRKY28 is able to bind to the *ICS1 *promoter *in vivo*, chromatin immunoprecipitation (ChIP) assays were set up using Arabidopsis protoplasts, as described by [33]. The *WRKY28 *coding sequence was fused to a haemagglutinin (HA) tag and expressed in Arabidopsis protoplasts. The resulting WRKY28-HA fusion protein was able to induce GUS expression when cotransfected with an *ICS1 promoter::GUS *construct, indicating that the HA tag did not interfere with WRKY28's functionality (Results not shown).

**Figure 7:**
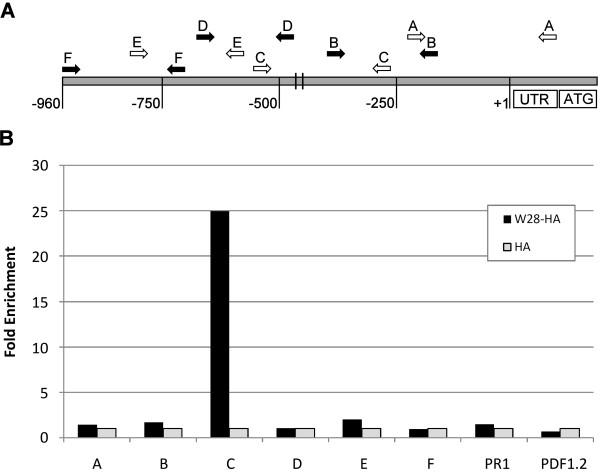
**Chromatin Immunoprecipitation assay of WRKY28**. Schematic representation of the location of primers corresponding to regions of the *ICS1 *gene used in the ChIP assays (A). Fold enrichment of immunoprecipitated DNA from protoplasts expressing WRKY28-HA versus protoplasts expressing unfused HA corrected for the qRT-PCR amplification efficiencies (B). The position of the WRKY28 binding sites at -445 and -460 is indicated.

For ChIP analysis WRKY28-HA or unfused HA were expressed in protoplasts. After 24 h incubation, chromatin complexes were cross-linked using formaldehyde. Upon exhaustive shearing by sonication, the fragmented chromatin was incubated with monoclonal anti-HA antibodies overnight, after which immunoprecipitated complexes were captured using magnetic protein G beads. DNA eluted from the beads was analyzed by qPCR with primers corresponding to six overlapping regions of the *ICS1 *promoter (Figure 7A). qPCRs with primers corresponding to the coding region of *PR1 *and the promoter region of *PDF1.2 *were included as controls. The results are shown in Figure 7B. With the primer sets corresponding to *PR1 *and *PDF1.2 *no specific products were amplified, indicating that these sequences were absent from the immunoprecipitated chromatin. While no specific PCR products were amplified with primer sets A, B, D, E and F, it is evident that the region corresponding to the *ICS1 *promoter bordered by primers C was highly enriched in the immunoprecipitated chromatin from the WRKY28-HA transfected protoplasts (25-fold in comparison to the control). This region contains the two WRKY28 binding sites at -445 and -460 as determined by EMSA (Figure 4A). A similar result was obtained with a primer pair covering a smaller region containing the two binding sites (Results not shown). In conclusion, the ChIP assays indicated that WRKY28 specifically binds to the *ICS1 *promoter *in vivo*, most probably to one or both binding sites at position -460 and -445 upstream of the transcription start site.

## Discussion

### WRKY28 and WRKY46 Activate Expression of *ICS1 *and *PBS3*, Respectively

Our *in silico *co-expression analysis of Arabidopsis transcription factor genes and genes involved in stress signaling suggested many putative new components of the signal transduction pathways [28]. Among the genes resulting from this screening were two encoding WRKY transcription factors linked to genes involved in SA metabolism. The gene encoding the WRKY type II member WRKY28 was found to be closely co-regulated with the *ICS1 *gene involved in SA biosynthesis, whereas the type III *WRKY46 *gene linked to *PBS3*. Based on this finding we decided to investigate the effects of these WRKYs on transcriptional activation of *ICS1 *and *PBS3*. Indeed, overexpression of WRKY28 in Arabidopsis protoplasts led to enhanced GUS activity from a co-expressed *GUS *reporter gene under control of a 1 kb *ICS1 *promoter, and also expression of the endogenous *ICS1 *gene was increased (Figures 1 and 2). Likewise, overexpression of WRKY46 resulted in increased accumulation of *PBS3 *mRNA, supporting the notion that WRKY46 is a transcriptional activator of *PBS3 *(Figure 2). GUS activity was not enhanced from a co-expressed 1 kb *PBS3 *promoter*::GUS *gene. This suggests that WRKY46 may activate the *PBS3 *gene by binding at a position in the promoter further upstream than 1 kb. However, we cannot exclude the possibility that the 1 kb promoter used for the construction of the reporter construct and which was derived from curated genome sequence data by The Arabidopsis Information Resource (TAIR), is not the actual *PBS3 *promoter. A detailed analysis of the region upstream of the coding sequence in the Arabidopsis genome shows that the intron of almost 1 kb suggested to be present in the 5'-UTR of *PBS3 *contains several putative binding sites for transcription factors like WRKYs and TGAs. It will be interesting to investigate if the suggested "intron" is the actual *PBS3 *promoter.

Functional analysis that would further support the important role of WRKY28 in *ICS1 *gene expression were hampered by the lack of WRKY28 knock-out mutants or T-DNA insertion lines, while our efforts to achieve silencing of WRKY28 through Agrobacterium-mediated transformation with pHANNIBAL constructs via flower dip only resulted in seedlings that died shortly after germination. These findings suggest that WRKY28 also plays an essential role during early plant development.

### DNA Binding Site of WRKY28

Several studies on DNA binding characteristics of WRKY transcription factors have led to the generally accepted consensus binding sequence TTGAC[C/T], commonly referred to as the W-box [25,30,34-39]. Recently, we identified a variant binding site for the tobacco NtWRKY12 transcription factor [31]. NtWRKY12 binds to a WK-box (TTTTCCAC), which deviates significantly from the W-box consensus sequence.

In this study we have characterized two sites in the *ICS1 *promoter that have a high affinity for WRKY28. The consensus WRKY28 binding site that emerged from this analysis has some characteristics that differ from the W-box consensus (Figure 5C). We found that, unlike the consensus W-box, a C may be present at position -1 in front of the TGAC core, and although a T is also allowed at -1, a C is then required at -2. Similarly, for the sequence after the core, in one of the binding sites an A is present at +1, which in the W-box is usually either a C or a T.

To disable binding of WRKY28 to the 30-bp EMSA probe harboring the binding sites at -460 and -445, mutation of both these sites was necessary. With only one site intact, binding was still possible (Figure 4A, Lanes 4 and 6). Nevertheless, with the 1 kb promoter, mutation of only one of the sites had a severe effect on reporter gene expression and expression was not further reduced when both sites were mutated. Apparently, for transcriptional activation both sites are required. Possibly, activation requires that WRKY28 binds as a dimer, similar to WRKYs 18, 40 and 60, which were found to form functionally relevant homo- and heterodimers [40].

The transactivation experiments also showed that mutation of the sites at -460 (m1) and -445 (m2) did not completely knock out reporter gene expression. In comparison to the GUS activity obtained with the wild type construct, approximately 20% remained. Furthermore, the reduction in basal expression levels seen with the mutant *ICS1 *promoters in the absence of overexpressed WRKY28 indicates that also endogenous factors binding to the sites at -460 and -445 contribute to the expression level. qRT-PCR has shown that the WRKY28 gene is much higher expressed in protoplasts than in suspension cells from which the protoplasts were made (Results not shown), suggesting that possibly these factors include endogenous WRKY28. Besides the direct activation of *ICS1 *gene expression, WRKY28 might also indirectly effect the *ICS1 gene *via transcriptional activation of genes encoding other transcription factors acting on the *ICS1 *promoter. Moreover, the residual GUS expression remaining with the m1, m2 and m1+2 mutant promoters could indicate that other sites in the ICS1 promoter are still able to bind WRKY28, although the existence of such sites was not supported by the results of the ChIP analysis.

## Conclusions

### Integrated Model for Regulation of SA Biosynthesis by WRKY28 and WRKY46

The combined results of the work described here, lead us to propose the following model for the induction of SA biosynthesis upon pathogen attack. Induction of the basal defense response starts with the detection of a pathogen-associated molecular pattern (PAMP), like in the case of flagellin, which is perceived by the FLS receptor. The activated FLS receptor triggers a MAP kinase cascade (MAPKKK/MEKK1?, MKK4/5, MPK3/6), which leads to transcriptional activation of the *WRKY28 *gene [26]. Transcription factor WRKY28 subsequently activates directly, and likely also indirectly via yet unknown transcription factors, expression of the *ICS1 *gene, through binding the promoter at the two binding sites at -460 and -445 and possibly at other sites, resulting in synthesis of ICS that catalyzes SA production. How the activated MAP kinase induces *WRKY28 *gene expression remains a matter of speculation. The activated MAPK could activate an as of yet unknown transcription factor on standby or release one from a repressor complex, or it may function itself as activator of *WRKY28 *expression.

**Figure 8:**
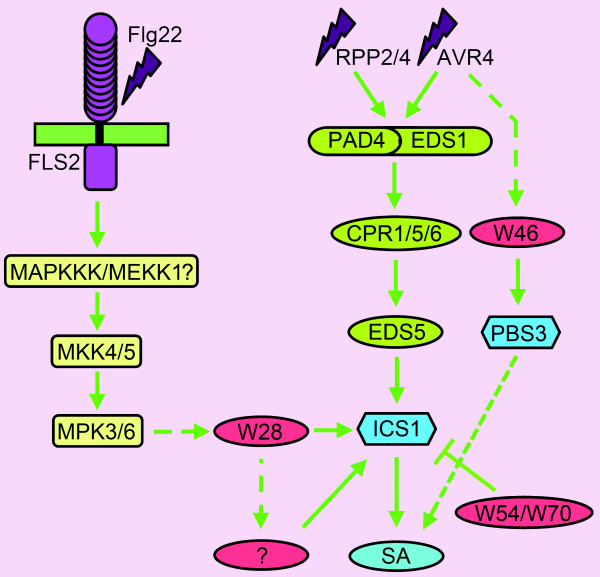
**Model for regulation of SA biosynthesis by WRKY28 and WRKY46**. Upon infection with a pathogen expressing flagellin (Flg22) or avirulence genes (RPP2/4 or AVR4), *WRKY28 *or *WRKY46 *are rapidly induced. Activation of FLS2 receptor by Flg22 results in activation of a MAPK cascade, which leads to induction of *WRKY28 *expression, which subsequently activates directly and likely also indirectly via yet unknown transcription factors (?), *ICS1 *gene expression leading to SA production. Avirulence factors like AVR4 trigger SA production through a pathway involving genes *PAD4*, *EDS1*, *CPR1/5/6*, *EDS5 *and *ICS1*. WRKY46 is rapidly synthesized and either directly or indirectly positively regulates *PBS3 *gene expression, having a positive influence on SA metabolism.

Less is known about the role of the product of the *PBS3 *gene. It is rapidly induced in plants recognizing pathogens carrying virulence factors, like in the case of *Pseudomonas syringae *containing AVR4 [27]. A function in SA metabolism has been suggested based on its effect on SA-glucoside accumulation and its similarity to phytohormone-amino acylases [22,23]. *PBS3 *gene expression is repressed by high levels of SA, indicating that it is more likely that PBS3 functions early in the defense response before SA levels start to rise [24]. Similarly, WRKY46 expression is rapidly induced upon infection and our finding that it enhances *PBS3 *gene expression suggests an early role in R-gene-mediated defense. Figure 8 shows the placement of the two WRKYs in the SA-signaling pathways.

## Methods

### Protoplast Preparation, Transfection and Analysis

For transactivation and qRT-PCR experiments, protoplasts were prepared from cell suspensions of *Arabidopsis thaliana *ecotype Col-0, according to van Verk *et al. *[31].

For transactivation experiments protoplasts were co-transfected with 2 μg of plasmids carrying reporter gene constructs *ICS1 promoter::GUS *(promoter refers to bp -1 to -960, relative to the transcriptional start site), or *PBS3 promoter::GUS *(promoter refers to bp -1 to -1000, relative to the transcriptional start site) and 6 μg of effector constructs *35S::WRKY28*or *35S::WRKY46 *in expression vector pRT101. As a control, cotransfection of promoter*::GUS *constructs with the empty expression vector pRT101 was carried out. The protoplasts were harvested 16 hrs after transformation and GUS activity was determined [41]. GUS activities from triplicate experiments were normalized against total protein level.

To analyze effects on expression of endogenous genes by WRKY28 and WRKY46, protoplasts were transfected with 6 μg of *35S::WRKY28 *or *35S::WRKY46 *expression plasmids. After 24 h protoplasts were harvested and total RNA was isolated. RNA was treated with DNAse using the Turbo DNA-free kit (Ambion) and cDNA was synthesized using the universal first strand cDNA synthesis kit (Fermentas). Expression of endogenous genes was determined by qPCR using primers listed in Table 1. qPCR was performed using a standard Phusion high fidelity polymyerase (Finzymes), supplemented with 0.145 μl Tween-20, 1.45 μl glycerol, 1 mM MgCl_2 _and 1× SybrGreen (Roche #70140720) per 50 μl reaction. The reactions were analyzed using a BioRad Chromo4 qPCR machine. MIQE data has been added as Additional File 1.

**Table 1:** Oligonucleotides used for qRT-PCR and ChIPqPCR analysis

qPCR-*Actin 3*	F	5'-CCTCATGCCATCCTCCGTCT-3'
	
	R	5'-CAGCGATACCTGAGAACATAGTGG-3'
qPCR-*Actin 7*	F	5'-AGTGGTCGTACAACCGGTATTGT-3'
	
	R	5'-GAGGAAGAGCATACCCCTCGTA-3'

qPCR-*Actin 8*	F	5'-AGTGGTCGTACAACCGGTATTGT-3'
	
	R	5'-GAGGATAGCATGTGGAAGTGAGAA-3'

qPCR-*β-Tubelin*	F	5'-GGAAGAAGCTGAGTACGAGCA-3'
	
	R	5'-GCAACTGGAAGTTGAGGTGTT-3'

qPCR-*ICS1*	F	5'-GGAACAGTGTCATCTGATCGTAATC-3'
	
	R	5'-CATTAAACTCAACCTGAGGGACTG-3'

qPCR-*PBS3*	F	5'-CGTACCGATCGTGTCATATGAAG-3'
	
	R	5'-CTTCACATGCTTGGTTATAACTTGC-3'

qPCR-*WRKY28*	F	5'-CAAGAGCCTTGATCGATCATTG-3'
	
	R	5'-GCAAGCCCAACTGTCTCATTC-3'

qPCR-*WRKY46*	F	5'-CATGAGATTGAGAACGGTGTG-3'
	
	R	5'-CTGCCATTAAGAGAGAGACATTACATTC-3'

ChIP-A	F	5'-GTCAAAGCTTGCACGACTAACTTTAGAAAAATG-3'
	
	R	5'-CAGTGGATCCTGCAGAAATTCGTAAAGTGTTTC-3'

ChIP-B	F	5'-GTCAAAGCTTCAACCAAACGAATCCGGTCTGT-3'
	
	R	5'-GAAGAGATCTATTTCATTTTCACACAAAATTTCTC-3'

ChIP-C	F	5'-GTCAAAGCTTCAAACGAGAAGAGTCGTCTAGC-3'
	
	R	5'-GGGTCAGTTAATTGTTTGATCTATTATTATTAG-3'

ChIP-D	F	5'-GTCAAAGCTTGCCATATGCCTTATGTACGAGA-3'
	
	R	5'-AGAAAGATCTTAGTGTAAAATTGCATAGACCAAG-3'

ChIP-E	F	5'-GTCAAAGCTTCTATGCTTTGTTTTACATGTAAAG-3'
	
	R	5'-GGGAAAAACATTACATGTCACTACAAATTGCAA-3'

ChIP-F	F	5'-GTCAAAGCTTCTGGTCTCAAAGAGCCTAAGTG-3'
	
	R	5'-GGGCTCCTTTAAATTTTGACACATTTCTAAAAT-3'

ChIP-*PR1*	F	5'-GTTCTTCCCTCGAAAGCTCAAGAT-3'
	
	R	5'-CACCTCACTTTGGCACATCCG-3'

ChIP-*PDF1.2*	F	5'-TATACTTGTGTAACTATGGCTTGG-3'
	
	R	5'-TGTTGATGGCTGGTTTCTCC-3'

### Electrophorectic Shift Assays

Protein for EMSAs was purified from *E. coli *transformed with pGEX-KG constructs containing the open reading frame of *WRKY28 *cloned in frame behind the GST open reading frame, according to van Verk *et al. *[31].

EMSAs were performed essentially as described by Green *et al. *[42]. DNA probes for the EMSA assays were obtained by slowly cooling down mixtures of equimolar amounts of complementary oligonucleotides with a 5'-GGG overhangs from 95°C to room temperature. Annealed oligonucleotides were subsequently end-filled using Klenow fragment and [α-^32^P]-dCTP, after which unincorporated label was removed by Autoseq G-50 column chromatography (Amersham-Pharmacia Biotech). EMSA reaction mixtures contained 0.5 μg purified protein, 3 μL 5× gel shift binding buffer [20% glycerol, 5 mM MgCl2, 2.5 mM EDTA, 2.5 mM DTT, 250 mMNaCl, 50 mMTris-HCl, pH 7.5, 0.25 mg mL^-1^poly(dI-dC) x poly(dIdC) (Promega)] in a total volume of 14 μL. After 10-min incubation at room temperature, 1 μL containing 30,000 cpm of labeled probe, representing approximately 0.01 pmol, was added and incubation was continued for 20 min at room temperature. Fifty- and 250-fold molar excess of unlabelled annealed oligonucleotides were added insome reactions as competitor. The total mixtures were loaded onto a 5% polyacrylamide gel in Tris-borate buffer and electrophoresed. After electrophoresis, the gel was dried, autoradiographed, and analyzed using X-ray film.

### Chromatin Immunoprecipitation

For ChIP assays, protoplasts were prepared as described above and transfected with 6 μg of *35S::WRKY28-HA *or *35S::HA *constructs in plasmid pRT101. After 24 h, protoplasts were harvested and ChIP assays were conducted as described by [33], with minor modifications. After formaldehyde fixation, the chromatin of the protoplasts was isolated and extensively sheared by sonication to obtain fragment sizes between 300-400 bp. Rat anti-HA monoclonal antibodies (clone 3F10, Roche) and Dynabeads Protein G magnetic beads (Invitrogen) were used to immunoprecipitate the genomic fragments. qPCRs were performed on the immunoprecipitated DNA using primer sets corresponding to six overlapping regions of the *ICS1 *promoter as shown in Figure 8A, and were corrected for their individual PCR amplification efficiencies. qPCRs with primers specific for the coding region of the *PR1 *gene and the promoter of *PDF1.2 *gene of Arabidopsis were used as controls. The primers used for the ChIP assays are listed in Table 1.

## Authors' contributions

MVV designed the study, carried out the analysis, helped in data interpretation, and made the draft of the manuscript. JFB and HL designed the study, helped in data interpretation, and edited the manuscript. All authors have given final approval for this version to be published.

## Supplementary Material

Additional file 1 **MIQE information for the qPCR experiment**. Click here for file
