# Supplementary material for: WRKY Transcription Factors Involved in Activation of SA Biosynthesis Genes
Source: BMC Plant Biol. 2011 May 19;11:89. doi: 10.1186/1471-2229-11-89 (PMC3120740; doi:10.1186/1471-2229-11-89)
Supplement: Additional file 1 — MIQE information for the qPCR experiment. [file 1471-2229-11-89-S1.DOC]

| **EXPERIMENTAL DESIGN** |
| --- |
| Definition of experimental and control groups |
| Number within each group |
| Assay carried out by core lab or investigator's lab? |
| Acknowledgement of authors' contributions |
| **SAMPLE** |
| Description |
| Volume/mass of sample processed |
| Microdissection or macrodissection |
| Processing procedure |
| If frozen - how and how quickly? |
| If fixed - with what, how quickly? |
| Sample storage conditions and duration (especially for FFPE samples) |

Experimental design is provided in the material and method section. RNA was isolated from three different experiments performed in different weeks at each condition. The control (non-induced) was generated by transfecting protoplasts with an empty-vector. Assays were carried out in the investigator’s lab.

In general, about 3 ml of protoplasts suspension was harvested per treatment, containing 0.5 million protoplasts, and directly frozen in liquid nitrogen after which RNA was isolated.

| **NUCLEIC ACID EXTRACTION** |
| --- |
| Procedure and/or instrumentation |
| Name of kit and details of any modifications |
| Source of additional reagents used |
| Details of DNase or RNAse treatment |
| Contamination assessment (DNA or RNA) |
| Nucleic acid quantification |
| Instrument and method |
| Purity (A260/A280) |
| Yield |
| RNA integrity method/instrument |
| RIN/RQI or Cq of 3' and 5' transcripts |
| Electrophoresis traces |
| Inhibition testing (Cq dilutions, spike or other) |

The RNA was isolated using phenol/chloroform extraction, and LiCl precipitation. All following procedures were performed according to the manufacturer’s instructions. To remove any remaining DNA traces, RNA was treated with DNAse using the Turbo DNA-free kit (Ambion) in a 10 µl volume. Contamination was assessed by a no RT control in the qPCR reaction.

The precipitated RNA was resuspended in water. A sample of 1 µl was measured with the Nanodrop from Thermo. The A260/280 and 260/230 ratio was above 2.0 for all samples. The yield is about 10-15 µg of RNA per 3 ml culture.

RNA integrity (RIN/RQI or Cq of 3' and 5' transcripts, Electrophoresis traces) and inhibition testing was not performed. However, the RNA samples were visually checked on a 2% agarose gel, and a DNA sample was similarly treated with Phenol and Chloroform and was compared to untreated DNA sample with no difference in the Cq value, to validate the extraction method.

| **REVERSE TRANSCRIPTION** |
| --- |
| Complete reaction conditions |
| Amount of RNA and reaction volume |
| Priming oligonucleotide (if using GSP) and concentration |
| Reverse transcriptase and concentration |
| Temperature and time |
| Manufacturer of reagents and catalogue numbers |
| Cqs with and without RT |
| Storage conditions of cDNA |

cDNA was synthesized using the universal first strand cDNA synthesis kit (Fermentas) using 1 µg of RNA. The synthesis was performed according to the manufacturer’s instructions.

For all cDNAs, the Cqs with RT are about ICS1 (27), PBS3 (28), WRKY28 (21), WRKY46 (22), Actin2 (20), Actin7 (21), Actin8 (21) and B-tubelin (23), without RT no amplification was detected. cDNA was stored in at -20°C.

| **qPCR TARGET INFORMATION** |
| --- |
| If multiplex, efficiency and LOD of each assay. |
| Sequence accession number |
| Location of amplicon |
| Amplicon length |
| *In silico* specificity screen (BLAST, etc) |
| Pseudogenes, retropseudogenes or other homologs? |
| Sequence alignment |
| Secondary structure analysis of amplicon |
| Location of each primer by exon or intron (if applicable) |
| What splice variants are targeted? |

Multiplex qPCR was not performed. Sequence accession numbers are

NM_117927.2 (WRKY28), NM_130204.2 (WRKY46), NM_202414.1 (ICS1), NM_121335.3 (PBS3), NM_180280.1 (Actin2), NM_121018.3 (Actin7), NM_103814.3 (Actin8), NM_128508.2 (B-tubelin). Amplicon length is included in table under qPCR validation. *In silico* screen were performed with NCBI Blast. Primer were designed in exons (where possible spanning an intron) or UTR regions close to the 3’end of the gene. No splice variants were targeted.

| **qPCR OLIGONUCLEOTIDES** |
| --- |
| Primer sequences |
| RTPrimerDB Identification Number |
| Probe sequences |
| Location and identity of any modifications |
| Manufacturer of oligonucleotides |
| Purification method |

| qPCR-*Actin 2* | F | 5'-CCTCATGCCATCCTCCGTCT-3' |
| --- | --- | --- |
|  | R | 5'-CAGCGATACCTGAGAACATAGTGG-3' |
| qPCR-*Actin 7* | F | 5'-AGTGGTCGTACAACCGGTATTGT-3' |
|  | R | 5'-GAGGAAGAGCATACCCCTCGTA-3' |
| qPCR-*Actin 8* | F | 5'-AGTGGTCGTACAACCGGTATTGT-3' |
|  | R | 5'-GAGGATAGCATGTGGAAGTGAGAA-3' |
| qPCR-*β-Tubelin* | F | 5'-GGAAGAAGCTGAGTACGAGCA-3' |
|  | R | 5'-GCAACTGGAAGTTGAGGTGTT-3' |
| qPCR-*ICS1* | F | 5'-GGAACAGTGTCATCTGATCGTAATC-3' |
|  | R | 5'-CATTAAACTCAACCTGAGGGACTG-3' |
| qPCR-*PBS3* | F | 5'-CGTACCGATCGTGTCATATGAAG-3' |
|  | R | 5'-CTTCACATGCTTGGTTATAACTTGC-3' |
| qPCR-*WRKY28* | F | 5'-CAAGAGCCTTGATCGATCATTG-3' |
|  | R | 5'-GCAAGCCCAACTGTCTCATTC-3' |
| qPCR-*WRKY46* | F | 5'-CATGAGATTGAGAACGGTGTG-3' |
|  | R | 5'-CTGCCATTAAGAGAGAGACATTACATTC-3' |

No modifications were used. Primers were purchase from Eurofins MWG Operon and are salt-free.

| **qPCR PROTOCOL** |
| --- |
| Complete reaction conditions |
| Reaction volume and amount of cDNA/DNA |
| Primer, (probe), Mg++ and dNTP concentrations |
| Polymerase identity and concentration |
| Buffer/kit identity and manufacturer |
| Exact chemical constitution of the buffer |
| Additives (SYBR Green I, DMSO, etc.) |
| Manufacturer of plates/tubes and catalog number |
| Complete thermocycling parameters |
| Reaction setup (manual/robotic) |
| Manufacturer of qPCR instrument |

Each qPCR reaction had a 50 µl reaction volume as described in the Methods section.

Tubes and lids were purchased from BIOPlastics (Cat# B72711 and B75701B)

Cycling parameters were:

98°C for 2 min,

98°C for 10 sec

57°C for 15 sec

72°C for 20 sec

Plate read

Cycle 39 times

Melting curve from 70°C to 95°C, read every 0.5°C, hold 0.5 sec

Reactions were set up manually in a designated room using designated equipment. qPCRs were performed with the BioRad Chromo4 qPCR machine.

| **qPCR VALIDATION** |
| --- |
| Evidence of optimisation (from gradients) |
| Specificity (gel, sequence, melt, or digest) |
| For SYBR Green I, Cq of the NTC |
| Standard curves with slope and y-intercept |
| PCR efficiency calculated from slope |
| Confidence interval for PCR efficiency or standard error |
| r2 of standard curve |
| Linear dynamic range |
| Cq variation at lower limit |
| Confidence intervals throughout range |
| Evidence for limit of detection |
| If multiplex, efficiency and LOD of each assay. |

The specificity of the amplification products have been confirmed by size estimations on a 1.5% agarose gel and by analyzing their melting curves. Without a template, no Cq could be determined since it never passed the threshold line. Up to 5% of a non-induced condition was analyzed during efficiency determination.

|  | **length (bp)** | **slope** | **y-intercept** | **% efficiency** | **r2** |
| --- | --- | --- | --- | --- | --- |
| **WRKY28** | 160 | -3.042 | 38.37 | 113 | 0.957 |
| **WRKY46** | 252 | -3.302 | 35.93 | 101 | 0.985 |
| **ICS1** | 241 | -3.428 | 36.52 | 96 | 0.994 |
| **PBS3** | 221 | -3.534 | 37.16 | 92 | 0.924 |
| **Actin 7** | 85 | -3.365 | 30.80 | 98 | 0.995 |
| **Actin 8** | 96 | -2.873 | 35.54 | 123 | 0.993 |
| **Actin 2** | 418 | -3.418 | 33.23 | 96 | 0.988 |
| **B-Tubelin** | 74 | -3.275 | 34.93 | 102 | 0.989 |

| **DATA ANALYSIS** |
| --- |
| qPCR analysis program (source, version) |
| Cq method determination |
| Outlier identification and disposition |
| Results of NTCs |
| Justification of number and choice of reference genes |
| Description of normalisation method |
| Number and concordance of biological replicates |
| Number and stage (RT or qPCR) of technical replicates |
| Repeatability (intra-assay variation) |
| Reproducibility (inter-assay variation, %CV) |
| Power analysis |
| Statistical methods for result significance |
| Software (source, version) |
| Cq or raw data submission using RDML |

qPCR analysis program (source, version): Biorad, Opticon 3

Cq’s were for all samples at once, per primer pair, set using the log scale to such a level that all reactions have an efficiency preferably between 90-110%. No data have been exclude from the calculations.

Results of NTCs: no amplification products present thus no Cqs.

Justification of number and choice of reference genes: for use with Arabidopsis samples we have previously good experience with those 4 reference genes

Description of normalization method: 4 endogenous reference genes

Number and concordance of biological replicates: 3

Number and stage (RT or qPCR) of technical replicates: 3 at qPCR level

Repeatability (intra-assay variation): on average within 0.25 cycle with a maximum of 0.5 - 1 cycles for all samples.
